# Is the proof in the PUDding? Reflections on previously undocumented data (PUD) in clinical competency committees

**DOI:** 10.1007/s40037-020-00621-0

**Published:** 2020-10-01

**Authors:** Daniel J. Schumacher, Benjamin Kinnear

**Affiliations:** 1 Department of Pediatrics, University of Cincinnati College of Medicine/Cincinnati Children’s Hospital Medical Center, Cincinnati, OH USA; 2 Department of Internal Medicine, University of Cincinnati College of Medicine, Cincinnati, OH USA

In the current issue of the journal, Tam and colleagues explore the use of previously undocumented data, or PUD, about resident performance in making assessment decisions at the level of the clinical competency committee (CCC) at a relatively small postgraduate subspecialty program [1]. Tam and colleagues define previously undocumented data as any information contributing to CCC discussions that was not in documentation brought to the meeting. They provide four categories of this data: summary impressions, contextualizing factors, personal anecdotes and hearsay. While others have described use of such data in CCCs, [2, 3] Tam et al. elaborate on reasons for using this data during CCC meetings and methods for managing it during discussions. The authors suggest that given current limitations of most programs of assessment, there are likely benefits of using previously undocumented data in CCCs to make decisions, and they advocate for this as an acceptable practice. They argue that this practice can help fill gaps in assessment data that are often lacking in quantity, quality or clarity [1]. We agree whole-heartedly that suboptimal assessment data is a major barrier to CCCs making optimal and defensible decisions [4, 5] and that a better understanding of previously undocumented data can help CCCs manage it during meetings. However, Tam and colleagues also acknowledge the potential limitations of their findings. Building on this, we believe four issues warrant further exploration: 1) use of previously undocumented data as a symptom of suboptimal programmatic assessment that perhaps should not be used to justify its routine use, 2) the role of program size in the study’s findings, 3) the potential introduction of bias created when using previously undocumented data, and 4) the likely range of trainee acceptance regarding previously undocumented data use.

Tam et al. note that multiple barriers exist to capturing previously undocumented data in formal documentation, such as documentation of “idiosyncratic experiences,” limited time, and challenges in capturing complex constructs (e.g. professionalism) or contextual factors [1]. Rather than working around these barriers by using more of this type of data, we believe these short-comings should provide a call to improve programmatic assessment. Filling these gaps should include emphasizing the power of snapshot assessments which document rich, subjective, idiosyncratic experiences [6, 7]. Systems should also be designed to provide time for meaningful, timely documentation of observations rather than making valuable assessments ferment in fallible memories before being poured out in CCC discussions. In short, we believe that while previously undocumented data will always exist, and likely provides value in CCC decision-making, it should not be used as a work-around for suboptimal programmatic assessment systems. Rather, it should drive improvement efforts.

The study presented by Tam et al. focuses on a program of 6–8 total learners, with a CCC comprising seven faculty members who have “typically …had the benefit of individual supervisory experience with each trainee ” [1]. In this setting, previously undocumented data may comprise observational data that simply was not documented, and hence is conceptually similar to usual programmatic assessment data. While this supports the use of such data for this program, this may not be transferrable to larger programs or programs in which CCC members have not worked directly with the learners being reviewed. Without direct observation of learner performance, previously undocumented data may be skewed toward hearsay rather than discussions of contextualizing factors, personal anecdotes (based on direct observation), and summary impressions (the four types of previously undocumented data Tam et al. describe). Thus, the conclusions that Tam et al. draw may be more appropriate for smaller sized programs where trainees and CCC members know each other well and work together regularly than for larger programs.

While all observational data carry some risk of bias, [8] use of previously undocumented data in a small group setting may increase this risk. Several cognitive biases can influence CCC decisions [9]. Use of previously undocumented data in these conversations may introduce or worsen availability bias, reliance on gist, selection bias, recency bias, recall bias, and visceral bias. Dickey et al. provide a caution in this regard, illustrating a faculty member “disregarding six months’ worth of data in favor of one recent patient interaction ” [9]. Allowing a small group such as a CCC to interject previously undocumented data also opens the door to other biases such as those based on race, gender or age. This brings the need for diverse CCC membership and robust faculty development on implicit bias into focus. With these considerations in mind, integrating this type of data requires careful consideration and attention toward whether this practice worsens biases potentially at play. Here again, program size may have an influence, and it may be particularly important in larger programs in which biased previously undocumented data from an individual CCC member could be either amplified or left unchallenged by another CCC member not familiar with the trainee.

Finally, the authors posit that trainees are likely to be accepting of previously undocumented data when receiving this information [1]. Here again, we wonder if program and faculty size may play an important role. If the trainees know all the members of the CCC well, have trusting relationships with most or all of them, and believe that the CCC members can characterize their performance accurately, we agree that presenting this type of data to trainees may not be problematic. However, does this hold true with larger programs where trainees may not know who sits on the CCC and may not have worked with multiple members previously? In this situation, trusting relationships built through personal experiences may be the exception rather than the rule, even for senior-level trainees. Would previously undocumented data be trusted by trainees or is there a risk that this data could be seen as hearsay, anecdotal one-offs, and unfair [10]? Further studies on learner perceptions of this type of data are needed.

Previous studies have considered the role previously undocumented data serves in CCC discussions, emphasizing this as a traditional practice that warrants continued study and even noting, similar to Tam et al., that such data may be helpful in filling gaps in documented assessment data [2, 3]. Tam and colleagues advance our understanding in this area, making an important contribution to the literature. However, we believe their study may also emphasize the importance of considering the transferability of research findings to other settings and wonder if their findings may represent smaller training programs better than larger ones. This has important implications for how previously undocumented data is used by CCCs and presented to trainees following CCC deliberations based on training program size if true. Perhaps most importantly, viewing Tam et al.’s findings through a lens of continual quality improvement provides an opportunity to use previously undocumented data as a driver to advance programmatic assessment.
